# Mobile phone screen protector glass: A TL investigation of the intrinsic background signal

**DOI:** 10.3389/fpubh.2022.969330

**Published:** 2022-09-15

**Authors:** Céline Bassinet, Michael Discher, Yoann Ristic, Clemens Woda

**Affiliations:** ^1^Institut de Radioprotection et de Sûreté Nucléaire, Fontenay-aux-Roses, France; ^2^Department of Environment and Biodiversity, Paris-Lodron-University of Salzburg, Salzburg, Austria; ^3^Helmholtz Zentrum München, Institute of Radiation Medicine, München, Germany

**Keywords:** retrospective dosimetry, radiological accident, emergency dosimeter, thermoluminescence, glass screen protector, mobile phones

## Abstract

Screen protector glasses are often used to protect the display screen surface of mobile phones against physical damage. Their dosimetric properties were recently studied by thermoluminescence with the aim of using these items as potential emergency dosimeters in the event of a radiological accident. They are sensitive to ionizing radiation and they could be easily removed and replaced without destroying the phone in case of a dose assessment. However, an intrinsic background signal that partially overlaps with the radiation-induced TL signal is observed. The reconstructed dose could be overestimated if not properly taken into account. The homogeneity of this confounding signal on the surface of several screen protectors was estimated and a chemical treatment with hydrofluoric acid (HF 40%) was tested to minimize its contribution. For most of the samples studied, the intrinsic background signal remained a serious issue for dose reconstruction. Additionally, the TL signals were measured in the red detector range using two different models of red-sensitive photomultiplier tubes. The homogeneity of the intrinsic background signal on the surface of screen protectors was examined and the results of the reduction of this signal by the chemical HF treatment were discussed.

## Introduction

Retrospective dosimetry methods are needed to rapidly and accurately assess the dose absorbed by the victims in the event of a radiological accident. During the last decade, the dosimetric properties of mobile phone materials (electronic components, display glass and touchscreen glass) have been extensively studied by luminescence methods (1) with the aim of using these items as emergency dosimeters. However, so far these methods frequently require destroying the phone. Thus, in case of a dose assessment, the device can no longer be used. Given the cost of modern phones and the need for communication in an emergency, this is a major issue in terms of acceptance by potentially overexposed people (1, 2). Alternative non-destructive approaches should be sought to overcome this problem.

The use of optically stimulated luminescence (OSL) from the protective back-glass found on modern mobile phones with wireless charging capabilities was recently proposed (3–5). A custom-made OSL reader was built to perform the measurements without dismantling the phone. Results of dose recovery tests on mobile phones protected from exposure to ambient light by their original opaque cases were promising. The use of OSL from camera lens glass protectors (6) was also investigated.

Another approach was proposed based on the mobile phone screen protector glasses. They have become very popular to protect the display screen surface against physical damage. This extra layer of material is placed on the touchscreen and could be easily removed and replaced without destroying the phone in case of a dose assessment. The dosimetric characteristics of screen protector glasses were investigated in a preliminary study (7) using thermoluminescence (TL) and a wideband blue detection window. A non-radiation induced signal (i.e., intrinsic background signal) which partially overlaps with the radiation-induced TL signal and is erased by the first TL measurement was observed for every screen protector glass. Its shape and intensity varied from screen protector to screen protector. The reconstructed dose could be overestimated if not properly considered. The presence of such a signal had also been reported for display glass (8, 9) and touchscreen glass (10). It came mainly from the glass surface layers. Methods had been proposed to reduce it using a mechanical treatment (11) by grinding the glass surface or a chemical treatment (12) by etching the glass with hydrofluoric acid (HF 40%). The screen protector glasses are too thin to use a mechanical treatment to try to reduce the contribution of the intrinsic background signal. The screen protectors would indeed be broken. The preliminary study (7) had shown a reduction in intrinsic background signal on screen protectors by etching their surface using HF, but the results were not properly quantified. The authors studied also in more detail the dosimetric properties of the screen protector glasses most sensitive to ionizing radiation and showed that the radiation-induced TL signal was sensitive to light. As the glass will frequently be illuminated by ambient light, this is a serious issue. Thus, TL measurements should be performed on the hard-to-bleach component of the TL signal, similar to LCD glasses (9). Systematic investigations on a larger set of screen protectors are needed to develop a robust measurement protocol, which is one of the goals of the joint ProGlaDos (Joining up to improve usage of mobile phone protective glass for retrospective dosimetry) research project. First, the optimization of the TL detection window was necessary to further investigate the dosimetric properties of screen protector glasses. This was achieved in this research project by systematically studying radiation-induced TL signals and intrinsic background signals (Discher, Bassinet, and Woda, A TL study of protective glasses of mobile phones for retrospective dosimetry, submitted) for several screen protectors using different filter combinations. It was impossible to identify a single detection window giving a sufficient radiation-induced signal and the lowest apparent dose due to the intrinsic background signal for all the glasses studied. However, a wideband detection window using Schott KG3 heat absorbing filters was recommended. This study also confirmed that an optical bleaching of radiation-induced TL signals should be carried out before their measurement. In a separate study, measurements of TL emission spectra indicated that for screen protectors a red emission exists, with potentially different properties regarding light sensitivity and fading compared to other emissions (Woda, Bassinet, and Discher, TL Emission spectra of screen protectors—implications for retrospective dosimetry, in preparation).

In the current work, the recommended detection window is used to study the intrinsic background signal of screen protectors in detail. The homogeneity of this intrinsic background signal on the surface of screen protectors is examined and the results of an attempt to minimize its contribution using chemical treatment are also presented. These measurements are carried out using a standard UV-VIS photomultiplier tube (PMT) with a sensitivity covering the wavelength range from UV to visible light, that is generally fitted in commercial luminescence readers. In addition, preliminary results obtained using two other PMT types with efficiencies extending to longer wavelengths, to selectively measure the red emission, are presented and discussed. The luminescence measurements were taken without delay after irradiation, fading not being considered in the present study. This parameter will be covered in other publications.

## Materials and methods

Mobile phone glass screen protectors of different brands (Belkin, Mobilis, Otter Box, ZAGG) suitable for different mobile phone models were chosen for this study. Information on these screen protectors given on the storage boxes of the screen protectors when purchased is provided in Table 1. One sample of each model was investigated except for P5. For this model, two samples were used, one to study the homogeneity of the intrinsic background signal on the glass surface and another for etching tests.

**Table 1:** Screen protectors used in this study.

**Sample ID**	**Brand**	**General information**	
		**Type of glass according to product description**	**Screen protector for mobile phone model**
P4	ZAGG	Invisibleshield glass, new ion matrix™ technology	Samsung galaxy J7
P5	Mobilis	Tempered glass, 9H hardness	Samsung galaxy Xcover4
P6	Otter box	Alpha Glass fortified glass	Apple iPhone 8 plus, 7 plus, 6s plus, 6 plus
P7	Belkin	Screenforce™ TemperedCurve	Samsung galaxy S9
P14	Belkin	Screenforce® TemperedGlass high-quality Japanese glass,	Apple iPhone 8, 7, 6s, 6
P18	Belkin	Screenforce™ InvisiGlass Ultra™ ion exchange strengthened	Apple iPhone Xs max
P20	Mobilis	Tempered glass, 9H hardness	Samsung galaxy A5
P21	Mobilis	Tempered glass, 9H hardness	Universal for smartphone 5.3–5.5″
P24	Belkin	InvisiGlass Ultra™ ion exchange strengthened glass real glass (100%)	Apple iPhone XR
P28	Belkin	TemperedGlass high-quality Japanese glass, 9H	Samsung galaxy A3

Screen protectors were cleaned with acetone and cut into pieces of ~5 × 5 mm^2^ to fit into the measurement cup of the luminescence reader. To attempt the intrinsic background signal reduction, the glass surface from screen protectors was etched using HF (40%) (12) for different etching times. After this chemical treatment, the samples were cleaned again with acetone. Three aliquots of each screen protector sample were measured for each etching time (from 1 to 5 min).

Luminescence measurements of the screen protector glasses were performed at IRSN (Fontenay-aux-Roses) with an automated Freiberg Instruments lexsyg smart system (13), equipped with a bi-alkaline Hamamatsu H7360-02 photomultiplier tube (300–650 nm). TL measurements were done on the hard-to-bleach component of the TL signal, after an optical bleaching of the signals. The bleaching time was 500 s using the blue LEDs (458 ± 5 nm) of the reader as a blue light source (7). The maximum power of the blue LEDs'unit is 100 mW/cm^2^, but only 90% of the maximum power was used for bleaching. For TL measurements, two Schott filters (KG3, each 3 mm thick, 290–890 nm) were used to define a wideband detection window. A second reading with the same parameters was done to subtract the thermal background. All TL measurements were carried out under a nitrogen atmosphere using a heating rate of 2°C.s^−1^ up to 400°C. TL signals were normalized to aliquot mass for comparison purposes. The luminescence reader is equipped with a built-in beta source (^90^Sr/^90^Y). All irradiations were carried out using this source. It was calibrated in air kerma from TL measurements of glasses (LCD and screen protectors) irradiated at the IRSN's linear accelerator (Elekta Synergy®) with a 4 MV photon beam. The dose rate of the beta source was ≈48 mGy.s^−1^. All luminescence measurements were taken directly after irradiation in order to avoid signal fading.

Additional luminescence measurements were carried out with two automated lexsyg research readers (Freiberg Instruments), located in the luminescence laboratories in Munich and Salzburg, which are equipped with red-sensitive PMTs (14). Measurements at Munich were carried out with a red enhanced UV/VIS Hamamatsu photomultiplier H7421-40 (300–720 nm), measurements at Salzburg with a VIS/NIR Hamamatsu photomultiplier tube H7421-50 (380 and 890 nm). Both PMTs are thermoelectrically cooled. Built-in ^90^Sr/^90^Y beta sources of both readers were calibrated for display glass using a standard ^137^Cs gamma source of the radiation facilities at HMGU (Buchler Kalibrator OB20), with dose rates of ≈58 mGy.s^−1^ and ≈20 mGy.s^−1^ for Salzburg and Munich, respectively. To enhance the detection of the red emission a combination of a Schott KG3 (3 mm) and a Schott OG570 (3 mm) longpass filter was used for TL measurements. All other measurement parameters were identical to the ones used at IRSN.

## Results

### Intrinsic background dose distribution over the glass surface

The intrinsic background dose distribution over the glass surface was investigated for four screen protectors of different brands (P4, P5, P6, and P7). The protectors were divided into several parts. Then, for each part, a glass aliquot was taken and its intrinsic background signal was measured. Then, each of these aliquots was irradiated at a calibration dose of 5 Gy and the TL signal measured.

**Figure 1:**
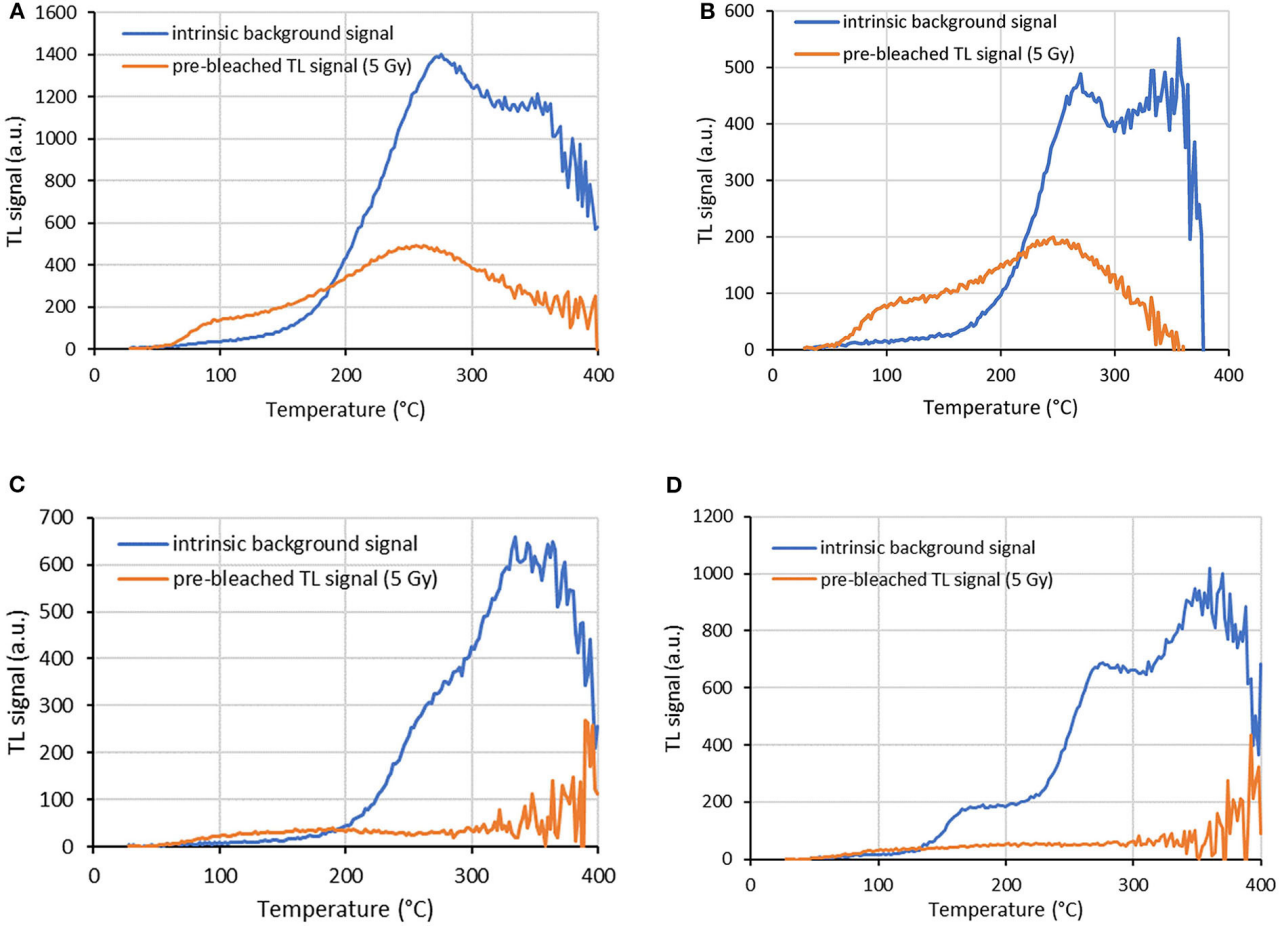
Intrinsic background signal of an aliquot of each screen protector investigated. Pre-bleached radiation-induced TL signal of 5 Gy is also shown. TL signals are normalized to aliquot mass. Screen protector ID: **(A)** P4, **(B)** P5, **(C)** P6, **(D)** P7.

The TL signals recorded for a representative aliquot of each screen protector studied are shown in Figure 1. The intensity of the radiation-induced TL signal (5 Gy) is slightly higher than that of the intrinsic background signal for temperatures below 200°C. The intrinsic background signal is predominant above this temperature and even from 150°C for P7.

**Figure 2:**
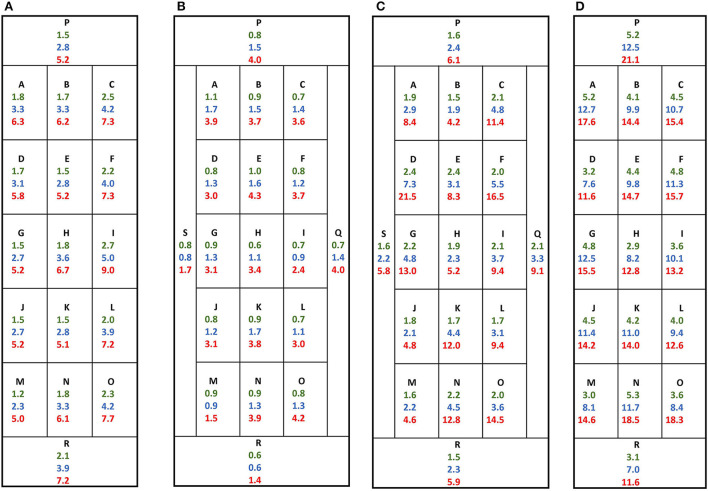
Intrinsic background dose distribution over the surface of four screen potectors. The corresponding intrinsic background doses for an aliquot of each part are given in Gy for three integration ranges (in green: 100–150°C, in blue: 100–200°C and in red: 100–250°C). Screen protector ID: **(A)** P4, **(B)** P5, **(C)** P6, **(D)** P7.

The integration range of the TL signals for the dose determination was generally chosen as a compromise between sufficient thermal stability, therefore integration always started at 100°C, and a minimized contribution from the intrinsic background signal (9). In order to estimate the influence of the latter, for the glass aliquot of each screen protector part the corresponding intrinsic background doses were calculated for three temperature intervals (100–150°C, 100–200°C, and 100–250°C) by comparing the intrinsic background signal with the 5 Gy signal. The variation of the intrinsic background signal on the surface of the screen protectors is presented in Figure 2. Mean intrinsic background doses and standard deviations of the aliquots are given in Table 2. As already observed (7), the intrinsic background dose is different from screen protector to screen protector. For the integration range 100–200°C for example, the mean intrinsic background doses and standard deviations are 3.4 ± 0.7 Gy, 1.3 ± 0.3 Gy, 3.5 ± 1.4 Gy, and 10.1 ± 1.8 Gy for P4, P5, P6, and P7, respectively. The intrinsic background dose increases when increasing the upper limit of the temperature integration range. For P4 for example, the mean intrinsic background doses and standard deviations are 1.8 ± 0.4 Gy, 3.4 ± 0.7 Gy, and 6.3 ± 1.2 Gy for the integration intervals 100–150°C, 100–200°C, and 100–250°C, respectively. However, the intrinsic background doses are quite homogeneously distributed over the glass surface of the screen protectors with relative standard deviations ranging from 15 to 28%, except for P6 and the highest integration ranges (41 and 48% for the integration intervals 100–200°C and 100–250°C respectively).

**Table 2:** Mean estimated intrinsic background doses and standard deviations (SD) on the surface of four screen protectors for three temperature intervals.

**Temperature interval (°C)**	**Mean estimated intrinsic background doses** ±**SD (Gy) for different screen protectors**			
	**P4**	**P5**	**P6**	**P7**
100–150	1.8 ± 0.4 (22%)	0.8 ± 0.1 (17%)	1.9 ± 0.3 (15%)	4.2 ± 0.8 (19%)
100–200	3.4 ± 0.7 (21%)	1.3 ± 0.3 (23%)	3.5 ± 1.4 (41%)	10.1 ± 1.8 (18%)
100–250	6.3 ± 1.2 (18%)	3.2 ± 0.9 (28%)	9.6 ± 4.6 (48%)	15.0 ± 2.6 (17%)

Relative SD are also given in brackets.

### Glass surface etching using a chemical treatment

In an attempt to reduce the contribution of the intrinsic background signal and optimize the integration range, a chemical treatment with HF (40%) was used (12) to etch the glass surface of the 10 screen protectors presented in Table 1. Glass pieces were etched for different times, from 1 to 5 min, depending on the screen protector model. This was necessary as the etching rate was highly variable between the different screen protector models. For example, samples from P18 and P24 screen protectors completely dissolved already after 2 min of treatment. For each screen protector, the intrinsic background signal of three aliquots per etching time was measured. Then, each aliquot was irradiated at 5 Gy and the TL calibration signal measured.

In the preliminary study (7), glasses could be grouped into two categories according to the sensitivity to radiation. The radiation sensitivity of glasses from category 1 was lower than that of glasses from category 2. Both categories are confirmed in the current study. The radiation-sensitivity of two glasses (P18 and P24) was much higher than that of the other samples (P4, P5, P6, P7, P14, P20, P21, and P28). P18 and P24 are screen protector glasses from Belkin made of InvisiGlass Ultra™.

**Figure 3:**
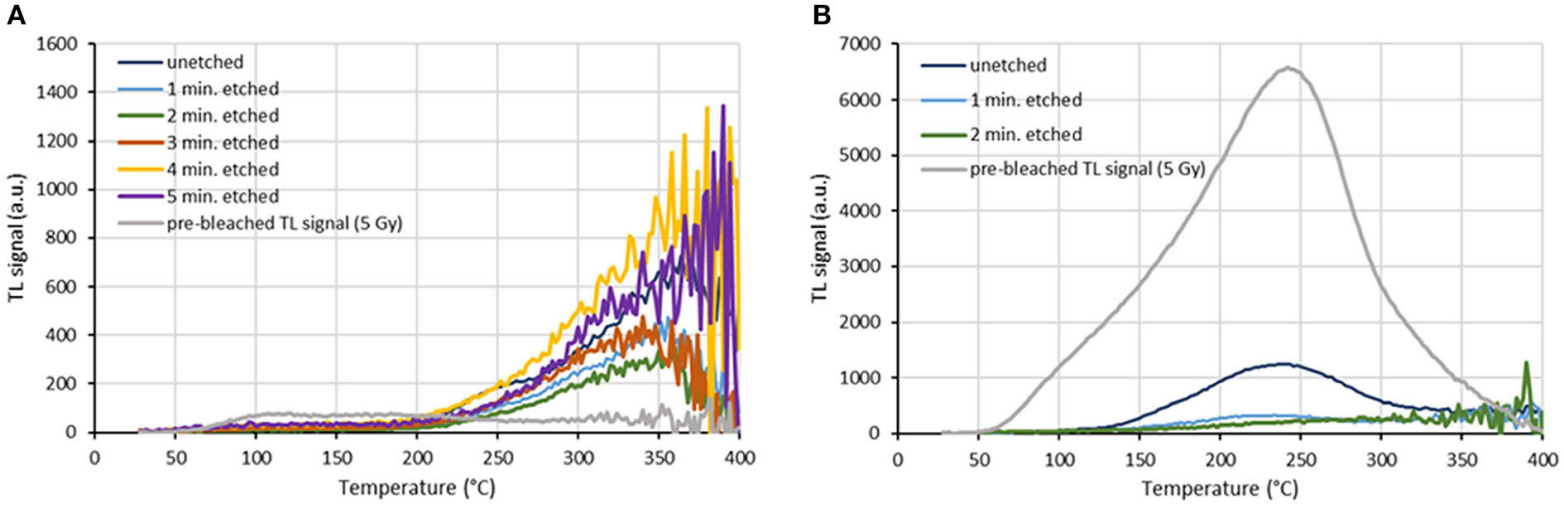
Variation of etching time for two screen protectors. The intrinsic background signals are shown for an aliquot per etching time. The intrinsic background signal of an unetched aliquot and the radiation-induced TL signal of this aliquot are also shown. TL signals are normalized to aliquot mass. Screen protector ID: **(A)** P21, **(B)** P18.

**Figure 4:**
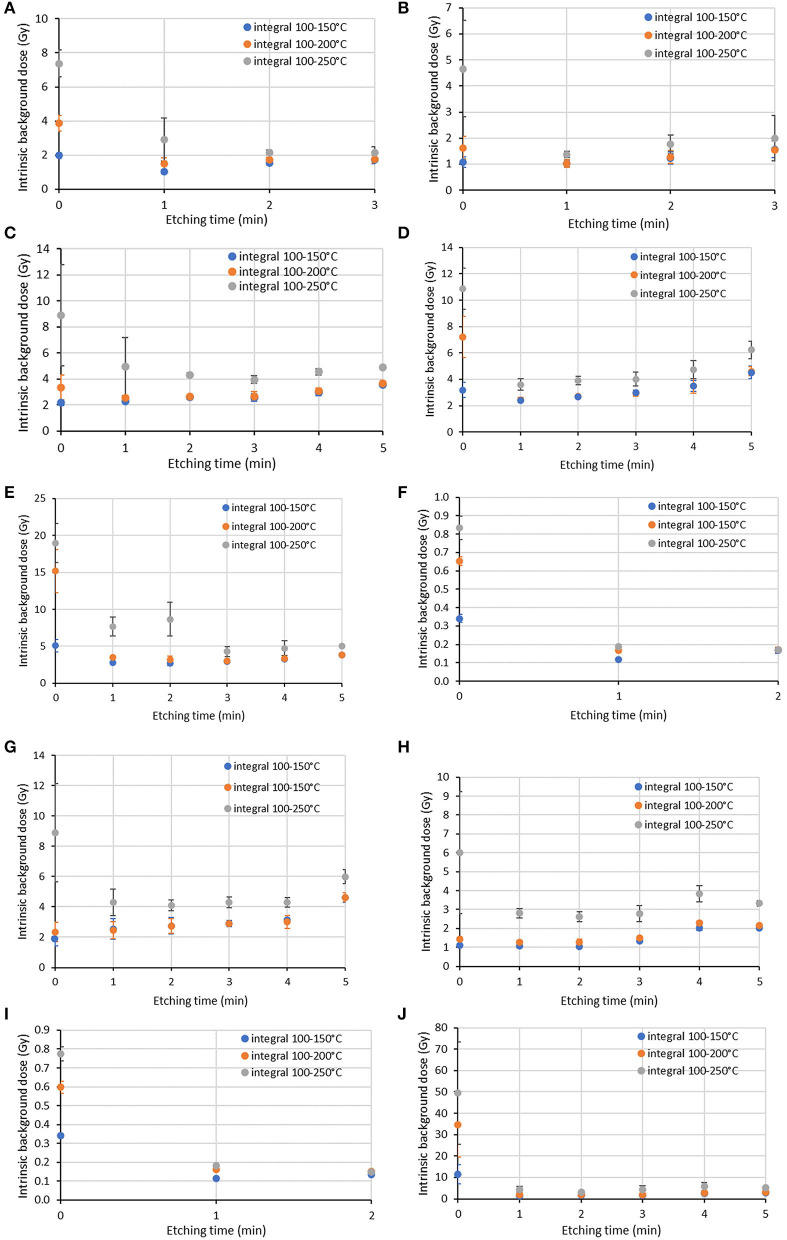
Variation of intrinsic background dose (Gy) with etching time for three integration ranges (100–150°C, 100–200°C, and 100–250°C). Mean and standard deviation as error bars for three aliquots are shown. Screen protector ID: **(A)** P4, **(B)** P5, **(C)** P6, **(D)** P7, **(E)** P14, **(F)** P18, **(G)** P20, **(H)** P21, **(I)** P24, **(J)** P28.

For a representative screen protector of each category, the intrinsic background signals measured for an aliquot per etching time are shown in Figure 3. The intrinsic background signal of an unetched aliquot and the radiation-induced TL signal of this aliquot are also shown. For each screen protector investigated, the variation of intrinsic background dose with etching time for three integration ranges (100–150°C, 100–200°C, and 100–250°C) is presented in Figure 4.

For P18 and P24, an intrinsic background dose below 1 Gy is observed for unetched aliquots for all integration ranges considered. The intrinsic background dose is reduced after an etching time of 1 min for all integration ranges. A longer etching treatment does not really improve the results and the intrinsic background doses converge at 0.1–0.2 Gy for all integration ranges investigated for these screen protectors. A temperature range of 100–250°C could be chosen for dose assessment for this type of glass.

For the other screen protector models investigated, an intrinsic background dose of a few gray is observed for unetched aliquots. For the integration ranges 100–150°C for example, it is in the range 1.1–2.2 Gy for P4, P5, P6, P20, and P21 and higher (3.2–11.5 Gy) for P7, P14, and P28. For all these screen protectors, the intrinsic background signal generally decreases after an etching time of 1 min and the intrinsic background dose falls into the ranges 1.0–2.8 Gy and 1.0–3.5 Gy for the integration ranges 100–150°C and 100–200°C, respectively. The improvement with increasing the etching time is generally not significant and could be affected by the mass normalization and its uncertainty. For the temperature range 100–250°C, the intrinsic background dose remains higher than for the other two integration ranges for most of these models.

As mentioned in the introduction, the red emission of screen protectors, identified in TL spectral measurements, could potentially have different dosimetric properties than the other emissions, therefore it was investigated, whether the intrinsic background doses could also be different when measuring in this wavelength range.

Three screen protectors (P4, P6, and P18) were investigated. For each screen protector and each measurement system, the intrinsic background signal of three unetched aliquots and three aliquots etched for 1 min using HF was measured. Then, each aliquot was irradiated at 5 Gy and the TL calibration signal measured. Mean intrinsic background doses and standard deviations of the aliquots are given in Table 3 for the three integration ranges used before (100–150°C, 100–200°C, and 100–250°C). For comparison, the results obtained using the standard UV-VIS PMT (see Figure 4) are also included. For sample P4 a significantly lower intrinsic background dose is obtained in the red detection window for unetched and etched aliquots in 75% of the cases, when using the standard error for comparison. For P6 this applies to all aliquots and integration windows. The reduction in intrinsic background dose can be almost up to an order of magnitude. For sample P18, which already showed low intrinsic background doses in the standard configuration, no improvement is obtained with the red detection window.

**Table 3:** Comparison of results obtained using three detection systems.

**Screen protector ID**	**Mean estimated intrinsic background doses** ±**SD (Gy) for different detection systems**		
	**Standard PMT**	**VIS/NIR PMT**	**VIS/Red enhanced PMT**
P4, unetched aliquots	2.0 ± 0.2 (9%)	1.4 ± 0.6 (41%)	1.1 ± 0.2 (14%)
	3.9 ± 0.5 (12%)	2.8 ± 0.6 (23%)	1.9 ± 0.3 (14%)
	7.4 ± 0.8 (11%)	5.2 ± 0.8 (16%)	3.8 ± 0.5 (13%)
P4, etched aliquots	1.0 ± 0.1 (12%)	0.3 ± 0.1 (54%)	0.5 ± 0.4 (84%)
	1.5 ± 0.4 (24%)	0.7 ± 0.0 (8%)	0.7 ± 0.4 (47%)
	2.9 ± 1.3 (43%)	1.2 ± 0.8 (68%)	1.0 ± 0.2 (21%)
P6, unetched aliquots	2.2 ± 0.2 (9%)	0.5 ± 0.3 (54%)	0.1 ± 0.2 (192%)
	3.3 ± 1.0 (29%)	0.6 ± 0.3 (51%)	0.5 ± 0.2 (49%)
	8.9 ± 3.9 (44%)	1.5 ± 1.6 (107%)	0.6 ± 0.4 (69%)
P6, etched aliquots	2.3 ± 0.0 (2%)	0.3 ± 0.1 (28%)	0.3 ± 0.4 (113%)
	2.5 ± 0.2 (7%)	0.5 ± 0.2 (43%)	0.5 ± 0.5 (90%)
	4.9 ± 2.2 (45%)	0.5 ± 1.2 (238%)	0.7 ± 0.5 (73%)
P18, unetched aliquots	0.3 ± 0.0 (6%)	0.4 ± 0.1 (14%)	0.5 ± 0.0 (8%)
	0.7 ± 0.0 (4%)	0.7 ± 0.1 (12%)	0.3 ± 0.0 (12%)
	0.8 ± 0.1 (8%)	0.9 ± 0.1 (11%)	0.8 ± 0.1 (7%)
P18, etched aliquots	0.1 ± 0.0 (3%)	0.1 ± 0.0 (10%)	0.1 ± 0.1 (64%)
	0.2 ± 0.0 (7%)	0.2 ± 0.0 (6%)	0.1 ± 0.0 (40%)
	0.2 ± 0.0 (5%)	0.2 ± 0.0 (17%)	0.1 ± 0.1 (70%)

For each screen protector (P4, P6, and P18), three unetched glass aliquots and three etched glass aliquots (HF, 1 min) were measured. Mean estimated intrinsic background doses and standard deviations (SD) are given in Gy for three integration ranges (in green: 100–150°C, in blue: 100–200°C and in red: 100–250°C). Relative SD are also given in brackets.

## Discussion

This study showed that for three out of four screen protectors investigated, the standard deviation of the distribution of the intrinsic background dose over the surface of the protector was below 30%. This value is somewhat higher than the one found by Discher and Woda (9) for the backside glass of a mobile phone display (10%) but can still be regarded as an acceptable level of homogeneity for retrospective dosimetry. Kim et al. (15) investigated the intrinsic background signal across the surface of AMOLED substrate glasses from mobile phone displays. High intrinsic background dose variation was observed for some samples.

A chemical treatment using HF was tested to reduce the intrinsic background signal of screen protectors in the temperature range that could be used for dose estimation. A decrease of the intrinsic background signal was observed after an etching time of 1 min. However, for most of the studied samples, this confounding signal remained high compared to the radiation-induced TL signal recorded after bleaching and the intrinsic background dose was higher than 1 Gy for the integration range 100–200°C. This will affect the detection limit of the screen protectors. Further data are needed to estimate it properly for screen protector glasses. Similar to the results of studies on display glass and touchscreen glass, the intrinsic background signal originates mainly from the glass surface and is probably due to glass additional treatments (e.g., anti-fingerprint and anti-scratch coating, privacy filters, etc.). These layers were partially removed using HF treatment. An analysis of the chemical composition of the screen protectors before and after HF treatment could be useful to confirm this hypothesis.

Mobile phone screen protectors in glass would be useful items for measuring the amount of dose an individual has received when personal dosimeters are not available. The present study represents one step in order to develop a robust measurement protocol for dose assessment in case of a radiological accident. For the moment, the intrinsic background signal and its variability between glass samples is the main limitation for using this dosimetry method in case of a radiological accident. An interesting observation in this context was the reduction of the intrinsic background dose when measuring at longer wavelengths (above around 600 nm) for two out of three samples. Further measurements on a larger set of screen protectors will be performed to confirm this observation. In addition, the intrinsic background dose may be regarded as the main limitation for the detection limit only if measurements are carried out immediately after irradiation or if the radiation-induced TL signal is thermally stable. In a real accident, hours, days or even weeks may pass between exposure and dose assessment. While little fading of the radiation-induced TL signal was observed for three screen protectors in a previous study (7), preliminary results on a larger dataset in a follow-up study showed that this is not always the case (Discher, Bassinet, and Woda, A TL study of protective glasses of mobile phones for retrospective dosimetry, submitted). For those screen protectors, where fading has a pronounced effect, the achievable detection limit will be influenced by both, the degree of fading and the intrinsic background dose. Both will depend on the choice of the temperature interval used for signal integration. Additional signal stability studies should thus allow to select an optimized temperature range for dose estimation.

## Data availability statement

The original contributions presented in the study are included in the article, further inquiries can be directed to the corresponding author/s.

## Author contributions

CB and YR: preparation of the samples. CB, MD, and CW: measurement, data analysis, interpretation, and manuscript preparation. All authors contributed to the article and approved the submitted version.

## Funding

The scientific cooperation was supported by the French Ministry of Foreign Affairs and the French Ministry of Higher Education and Research (project number: 46275WC) and by Scientific & Technological Cooperation (S&T Cooperation) grant, funded by funds of the Federal Ministry of Education, Science and Research (BMBWF) Austria (project ID: FR 12/2021).

## Conflict of interest

The authors declare that the research was conducted in the absence of any commercial or financial relationships that could be construed as a potential conflict of interest.

## Publisher's note

All claims expressed in this article are solely those of the authors and do not necessarily represent those of their affiliated organizations, or those of the publisher, the editors and the reviewers. Any product that may be evaluated in this article, or claim that may be made by its manufacturer, is not guaranteed or endorsed by the publisher.
